# 3D Printed Structures Filled with Carbon Fibers and Functionalized with Mesenchymal Stem Cell Conditioned Media as In Vitro Cell Niches for Promoting Chondrogenesis

**DOI:** 10.3390/ma11010023

**Published:** 2017-12-24

**Authors:** Josefa Predestinación García-Ruíz, Andrés Díaz Lantada

**Affiliations:** 1 Departamento de Biología Molecular, Universidad Autónoma de Madrid, 28049 Cantoblanco-Madrid, Spain; josefap.garcia@uam.es; 2 Product Development Laboratory, Mechanical Engineering Department, Universidad Politécnica de Madrid (UPM), C/ José Gutiérrez Abascal 2, 28006 Madrid, Spain

**Keywords:** tissue engineering, scaffolds for tissue repair, stem cell niches, cell culture niches, stem cell conditioned medium, carbon fiber, rapid prototyping, additive manufacture, computer-aided design & engineering

## Abstract

In this study, we present a novel approach towards the straightforward, rapid, and low-cost development of biomimetic composite scaffolds for tissue engineering strategies. The system is based on the additive manufacture of a computer-designed lattice structure or framework, into which carbon fibers are subsequently knitted or incorporated. The 3D-printed lattice structure acts as support and the knitted carbon fibers perform as driving elements for promoting cell colonization of the three-dimensional construct. A human mesenchymal stem cell (h-MSC) conditioned medium (CM) is also used for improving the scaffold’s response and promoting cell adhesion, proliferation, and viability. Cell culture results—in which scaffolds become buried in collagen type II—provide relevant information regarding the viability of the composite scaffolds used and the prospective applications of the proposed approach. In fact, the advanced composite scaffold developed, together with the conditioned medium functionalization, constitutes a biomimetic stem cell niche with clear potential, not just for tendon and ligament repair, but also for cartilage and endochondral bone formation and regeneration strategies.

## 1. Introduction

The behavior and fate of stem cells are not just dependent on genetic information, but are also regulated by other biochemical and mechanical cues and signals (epigenetic cues) which come from their micro-environment. This local micro-environment, which provides physical and chemical support and signals for survival and regulation, is commonly referred to as the stem cell niche, and plays a fundamental role in all strategies linked to the development of biomimetic tissue engineering constructs, mainly for bone, cartilage, ligament, tendon, and muscle repair using mesenchymal stem cells (h-MSCs) [1,2]. For adequately replicating the 3D stem cell niche, chemical components including cytokines, growth and trophic factors (generated by cells themselves or by their surrounding companions), and other chemical factors have to be taken into account. In addition, several physical forces from the surrounding environment, such as tensile and compressive stresses, vibratory excitations, fluid shear stresses, and even the presence of electromagnetic fields and gravitational forces have to be considered. Other elements, such as the existence of companion stem cells and the differentiated cell types from the adjacent tissue, clearly affect stem cell dynamics, overall behavior, and fate. Finally, the physical and chemical properties of the extracellular matrix, such as stiffness, porosity, viscosity, elasticity, morphology, roughness, surface topography and composition, help to orient cells and their mutual interactions and, consequently, regulate stem cell function [3,4].

In spite of the recent advances in the field of tissue engineering, repair, and regeneration, a universal methodology for the development of bioinspired, biomimetic, and biomechanical tissue engineering scaffolds, which may serve as substitutes for extracellular matrices (ECMs), is not yet available; this is, first of all, due to the complexity of biological materials and systems [5], but also due to all the possible design resources, manufacturing technologies, and related materials available, the results of which have not been systematically compared.

For instance, additive manufacturing technologies allow for the precise control of final geometries from the design stage; however, such designs are normally obtained by combining Euclidean-based (simple) geometries and the final result does not adequately mimic the complexity of biomaterials. On the other hand, scaffolds obtained by phase separation and more “traditional” processes typically lead to more biomimetic sponges—even though their final outer form and repeatability are more difficult to control—than the use of computer-aided strategies linked to rapid prototyping using additive processes. Therefore, further research is needed to address the advantages of combining different technologies [6] for manufacturing enhanced, even personalized, scaffolds for tissue engineering studies and extracellular matrices with global (outer) geometries defined as implants for tissue repair.

Furthermore, increasing data show that progenitor cell niche formation is absolutely needed for tissue development and repair [7]. Indeed, the niche composition and 3D structure play a vital role in stem cell state and fate. The niche is created by the specific combination of trophic factors, produced by progenitor cells to maintain the capability for tissue repair and regeneration, and by a specific extracellular matrix. Recent studies have helped to highlight the extreme relevance of the incorporation of adequate growth factors within the scaffold for promoting biological regulation, cell differentiation, angiogenesis, and final tissue viability [8,9,10]. Such inclusion of biochemical effects, derived from the incorporation of growth factors, adds additional uncertainties to the already complex interactions between the scaffolds’ structure, morphology, and mechanical properties. Consequently, studies addressing the synergies between ECMs and growth factors and their impact on tissue viability are needed in the quest for a general methodology for tissue engineering scaffold development.

The aforementioned tissue engineering challenges are even greater in applications aimed at articular repair, where several types of tissue (bone, cartilage, ligament, tendon, etc.) must be replaced, if possible, using a single multi-functional (and normally multi-material) scaffold capable of promoting cell adhesion, growth, migration, gene expression, and differentiation into different types of tissue. The need of relevant gradients of properties for the promotion of biomimetic properties, aimed at an improved repair, has led to the development of composite scaffolds using different approaches previously reviewed [11,12].

Focusing more specifically on the repair of tendons and ligaments, scaffold-based options clearly benefit from recent advances in the field of tissue engineering, as recently reviewed [13,14]. Regarding synthetic scaffolds, there are already several commercially available options, most of them employing aforementioned synthetic fibers knitted in the form of chords or membranes to provide a better three-dimensional extracellular matrix. The use of fibers helps to align the cells and promotes their aggregation in the form of aligned bundles of fibers, thus imitating the morphology of fibrillar tissues. Interesting options linked to the use of fibers, especially carbon and glass fibers, have been previously reported and show promising results regarding not just the repair of tendons and ligaments, but also that of cartilage and even of bone defects [15,16,17].

However, obtaining a real three-dimensional extracellular matrix using only fibers (either short or large), which are sometimes too flexible to withstand shear stresses and to maintain a desired three-dimensional geometry, and sometimes too rigid to adapt to the tissues being repaired, still remains a challenge. Regarding biological scaffolds, decellularized mammalian tissues, which can be implanted with or without growth factors, are already being used as biomimetic extracellular matrices, although their mechanical performance and sustainable (and economic) development are still unresolved issues.

In addition, in spite of the number of articular repair and regeneration solutions available, both synthetic and biological scaffolds can cause adverse responses, including host rejection, dilation, calcification, infection, chronic inflammation, and, in the more severe cases, even carcinogenesis. Inadequate biomechanical responses and failures due to fatigue or abrasion problems have been also reported in articular repair constructs focusing on the repair of tendons and ligaments [13,14]. Therefore, additional designs, materials, manufacture processes, and biointegration strategies need to be implemented and assessed, both in vitro and in vivo, towards an ideal and universal scaffold for articular research, repair, and regeneration.

In this study, we present a novel approach towards the straightforward, rapid, and low-cost development of biomimetic composite scaffolds aimed at articular repair. The system is based on additive manufacture (or 3D printing), in our case using selective laser sintering of polyamide powder, of a computer-designed lattice structure or framework, into which carbon fibers are subsequently knitted or incorporated. The outer and inner geometries, the porosity–density distribution, and even the values and distribution of mechanical properties can be controlled and tuned from the design stage, even in a personalized way and in accordance with the morphology of the tissue being repaired; this is thanks to the use of computer-aided design and modeling resources, which promote knowledge-based approaches. The 3D-printed lattice structure acts as the support and the knitted carbon fibers perform as the driving elements for promoting cell colonization of the three-dimensional construct.

A human mesenchymal stem cell (h-MSC) conditioned medium (CM)—in fact, a solution of growth factors generated by the own progenitor stem cells—is also used for improving the scaffold’s response and promoting cell adhesion, proliferation, and viability, when compared with the 3D-printed construct with the fiber filling without functionalization. Cell culture results, in which scaffolds became buried in collagen type II, provide relevant information regarding the viability of the composite scaffolds used and the prospective applications of the proposed approach. In fact, the advanced composite scaffold developed, together with the conditioned medium functionalization, constitutes a biomimetic stem cell niche with clear potential, not just for tendon and ligament repair, but also for cartilage and endochondral bone formation and regeneration strategies, as also discussed. The following section details the materials and methods used, before describing and analyzing main results and proposing future steps towards the concept of the universal scaffold for articular repair.

## 2. Materials and Methods

### 2.1. Computer-Aided Design Processes

In our case, computer-aided design of the different geometries was carried out with the help of NX-8.5 (Siemens PLM Solutions, Plano, TX, USA), mainly using combinations of parametric, spline-based, and matrix-based features, and Boolean operations, as well as using convergent/divergent trusses for the incorporation of gradual variations to the values of density and mechanical properties and for the promotion of biomimetic approaches.

**Figure 1:**
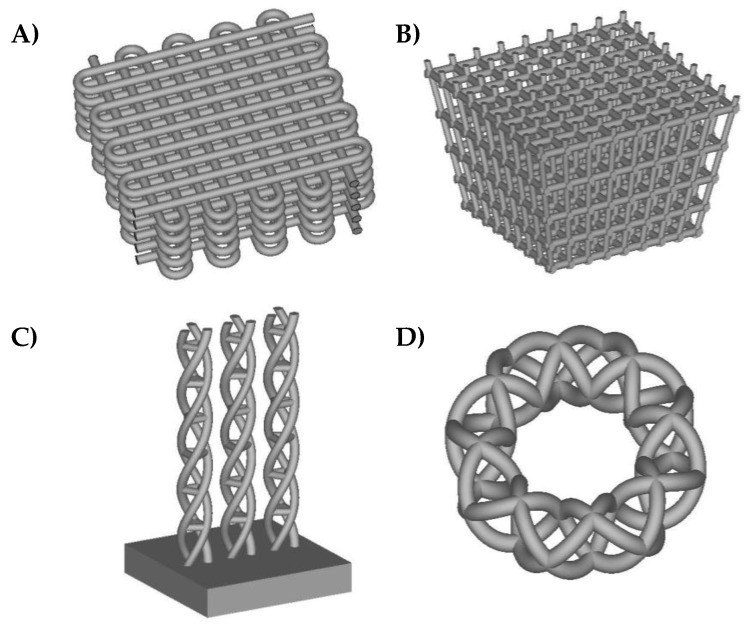
Computer-aided designs of lattice structures and frameworks, based on common tissue engineering scaffold geometries, for acting as supports for knitted carbon fibers. (**A**) Scaffold with porous geometry typically obtained by fused-deposition modeling; (**B**) Scaffold with functional gradient of porosity as potential bone repair model; (**C**) Tubular scaffold aimed at ligament and tendon repair; (**D**) Annular scaffold for sphincter tissue engineering.

Figure 1 shows different example geometries of biomimetic lattice structures and functionally graded scaffolds for subsequent solid freeform fabrication for potential articular repair strategies. In some of the different designs included, density and stiffness vary along their thickness, as happens in real living tissues. The spatial control of scaffolds’ properties is important for making cells grow in a similar environment as can be found in real organisms and for artificially obtaining final tissues with a biomimetic structure. For instance, in advanced bone tissue engineering, the transition between the trabecular and cortical regions is very interesting indeed. Therefore, scaffolds designed in a similar fashion as those from Figure 1 may prove useful in articular repair strategies, especially for the bony and chondral (axial skeleton) phases, as well as for fibrillar tissues.

The distances between pores and lattices are designed according to and taking into account the manufacturing precision of state-of-the-art rapid prototyping facilities working with parts in the mm^3^ range, which normally can achieve detail in the range of 400–500μm, as happens with the selective laser sintering technology used here for the manufacture of prototypes.

### 2.2. Rapid Manufacturing Processes

Rapid prototypes are obtained by means of additive manufacturing technologies, which allow for the manufacture of complex biomimetic geometries. For adequately carrying out the cell culture trials, the prototyping material, which is normally linked to the additive manufacturing technology used, must be taken into account. In our case, we obtain the prototypes in polyamide by resorting to state-of-the-art selective laser sintering. In selective laser sintering, the models are printed layer by layer, using a laser that draws thin lines upon polyamide powder. The laser melts and bonds the powder, so as to form a thin layer of the model. After a layer is printed, a new layer of fresh powder is spread over the surface by a roller. The printer has a print chamber that is heated to just below the melting point of the powder and the laser beam adds the extra energy to melt the powder, forming a solid model. For obtaining the prototypes in polyamide, we contracted the services of the 3D Print Lab of iMaterialise (http://i.materialise.com/, Leuven, Belgium).

**Figure 2:**
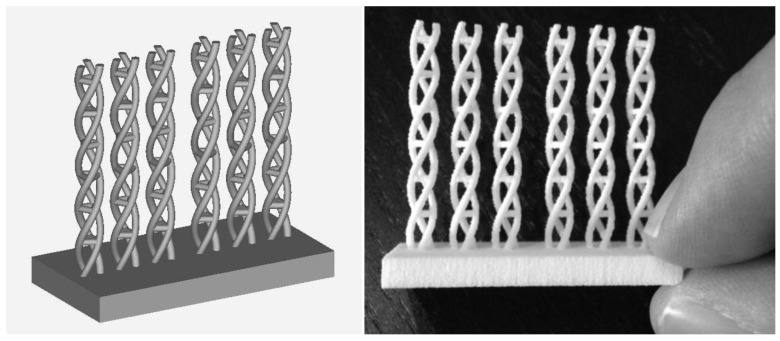
Selected computer-aided design of frameworks for knitting the carbon fibers and rapid prototype obtained by selective laser sintering of polyamide powder. For the math-based CAD processing of these helical biomimetic structures for fibrillar tissue repair, see previous work by our team [4].

Figure 2 shows the selected computer-aided design of frameworks for knitting the carbon fibers (obtained from a square of 10cm^2^ textured fabrics from Kaiyu, Hong Kong) and the rapid prototype obtained by selective laser sintering of polyamide powder (3D printing materials, iMaterialise, Materialise NV, Leuven, Belgium). Although the material is not commercialized as medical grade polyamide powder, alternative powders for additive manufacturing techniques by selective laser sintering may be used, which may constitute a topic for further research. It is necessary to highlight the complexity of the geometries attainable and the precision, which goes down to detail around 400microns, as further discussed in the following Section. After the manufacture of the frameworks, carbon fibers are knitted to them, just before their cutting and employment for the cell culture processes described further on, aimed at evaluating their potential as in vitro niches.

### 2.3. hMSCs Culture

The use of h-MSCs by García Ruíz and her related research projects were approved by the Spanish National Institute of Health and by the Madrid Autonoma University Ethic Committee to study the cellular and molecular bases of h-MSCs osteochondral differentiation, the mechanisms involved in age and degenerative diseases such as osteoporosis and related improving in biomaterials (approval document number CEI 58-1031, 6 October 2014). The hMSCs used were isolated from 1–2 mL taken from bone-marrow-derived samples from healthy donors, and were provided by Hospital La Princesa, the Jimenez–Diaz Foundation, and Malaga University Biobank. Cells were isolated and culture expanded following methods indicated in previous research [18,19].

In short, culture media DMEM-low glucose (LG), 0.25% Trypsin-EDTA, and Phosphate Buffered Saline (PBS) were prepared by the Research Service of the Molecular Biology Centre “Severo Ochoa”. Cells were seeded onto Falcon plates and expanded in DMEM-LG adjusted to 10% FBS of selected lots (Sigma, Madrid, Spain) to 80% confluence. Medium was replaced twice a week and cells were cultured until 70–80% confluence in a 5% CO_2_incubator (MRC, Iberlabo Spain) at 37 °C and 95% humidity. Osteochondral differentiation of hMSC was undertaken following the methodology used in previous works [18,20].

Cells were incubated in DMEM-LG with ITS (6.25 µg/mL insulin; 6.25 µg/mL transferrin; 6.25 µg/mL selenous acid) (Collaborative Research); 400µM AANE (nonessential amino acids, CBM), 2 mM pyruvate (Gibco), 37.5 µg/mL ascorbate (WAKO); 0.1mM dexamethasone (Decadran, Merck, Kenilworth, NJ, USA), and 0.06ng/mL TGF-b3 (R&D System, Minneapolis, MN, USA), during the time indicated. The medium was replaced twice per week.

### 2.4. hMSCs Conditioned Medium

For the preparation of each batch of CM-hMSCs, 8–10 p100 culture plates of hMSCs grown at 80% confluence in DMEM-LG and 10% FBS were used. Then, cells were washed thoroughly with PBS, incubated in DMEM-LG, starved of FBS, complemented with 2 mM pyruvate, and incubated during the following 24 h. Afterwards, the culture medium was collected, and cleaned of any floating cells in a bench centrifuge at 1500 rpm for 5 min.

As described in an earlier work [21,22], the clean supernatant was cooled down on ice for 30 min, centrifuged at 9000 rpm in a Sorvall centrifuge (Thermo Fisher Scientific, Waltham, MA USA) for a duration of 30 min to remove salt precipitations and, then, the clean supernatant was kept in 2 mL aliquots at −30 °C until use. We avoided repeatedly freeze–thawing the samples. The activity of each hMSC-CM batch was measured by triplicatedly measuring migration of individual cells in the leading edge of a scratch or wound-healing assay, using DMEM-LG–Pyr medium and DMEM-LG–10% FBS as controls.

### 2.5. Functionalization of 3D-Printed Structures Filled with Carbon Fibers

Carbon fibers were obtained from a square of 10 cm^2^ textured fabrics (Kaiyu, Hong Kong). A single bundle of carbon fibers was introduced into the 3D-printed structure with the help of a surgical clip. Then, the composites were exposed to the direct UV light of a cabin (Teltair) within culture plastic dishes.

**Figure 3:**
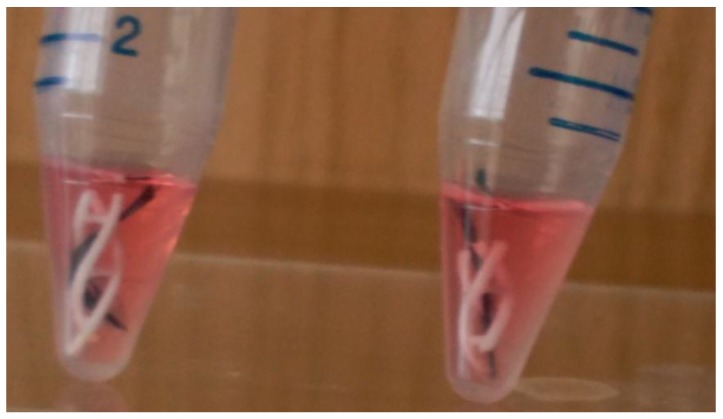
3D-printed lattices with embedded–knitted carbon fibers during the cell culture process.

The composite scaffolds were individually placed in 25 mL Falcon culture tubes(see Figure 3) and successively treated with 2 mL of (i) 95% ethanol, for a duration of 30 min and two times, to dissociate the bundle of carbon fibers into parallel carbon fibrils, with an average cross section of 4μm, by removing the holding resin used during fabric texturing; (ii) PBS wash; (iii) 2M HCl attack for 24 h for the acid activation of surfaces; (iv) PBS wash until neutralization; (v) surface coating CM-hMSCs for 24 h in cell incubator; (vi) removal of hMSC-CM and 10^5^ hMSCs seeding drop by drop. After 30 min in a cell incubator, the composite niches were exposed to a growth medium of DMEM-LG–10%FBS and to a chondrogenic differentiation medium, with composition described in [23,24], for a duration of seven days, changing the media twice a day.

### 2.6. Immunofluorescence

After cell culture, samples were processed as previously described [25,26]. Briefly, 3D composites were rinsed twice with ice-cold PBS and fixed in 3.7% formaldehyde in PBS for 30 min at room temperature, then washed in PBS and either used or kept at 4 °C. The cells present upon the 3D-printed polyamide lattice were detected by incubating with 0.1% crystal violet for 15 min, followed by removal of the dye excess, drying, and microscopy. The carbon fibrils of each composite sample were carefully removed out the niche and placed in individual wells of an M24 plate. Then, the cells on the carbon fibrils were incubated with 0.5% Triton X-100 in CSK (cytoskeleton) buffer containing 10 mM Pipes pH6.8, 3 mM MgCl2, 100 mM NaCl, 1 mM EGTA, 0.3M sucrose, and 1 mM PMSF for 30 min on ice to permeate and remove all cell-soluble proteins, fixed with 3.7% formaldehyde and washed with PBS, and kept at 4 °C until use for immune detection. For the former, cell preparations were blocked with 3% BSA–0.1% Triton X-100 in PBS (PBSA) for 2 h at room temperature and exposed to different antibodies. We used anti-α-tubulin (Sigma, Madrid, Spain) and anti-collagen type II (Invitrogen, Carlsbad, CA, USA). Secondary antibodies were labeled with either Alexa 488 or Alexa 594 (Invitrogen, by the Microscopy Service of MBC-CSIC-UAM) (Madrid, Spain). Nuclei were stained with DAPI (CALBIOCHEM) (Merck, Darmstadt, Germany) for 5 min. Immune stainings were observed with an inverted IX81 Olympus associated with a DP72 digital camera (Tokyo, Japan) and controlled by cellᴅ software.

## 3. Results

### 3.1. Design and Prototyping Results: Validation ofthe Development Process

The designs and prototypes obtained help to show that the manufacture of complex biomimetic and biomechanical lattice structures, porous geometries, and functionally graded materials—whose applications in the biomedical field are noteworthy—can be directly accomplished by using additive manufacturing technologies, typically working on a layer-by-layer approach (even though new advances are enabling the manufacture of several layers at once) and following the information regarding the three-dimensional geometries of parts and constructs stored in the form of CAD files.

By means of example, selective laser sintering enables the manufacture of frameworks, with detail down to 400–500 μm for parts in the mm^3^ range, using polyamide powder, with results adequate for in vitro cell culture trials as detailed in the following subsection, although the in vivo performance still needs to be assessed. Studying the combination of carbon fiber filler frameworks similar to the ones presented here, but manufactured using alternative technologies and materials, may help to systematically develop and assess the performance of a wide set of geometries and materials, as real 3D cell culture matrices, which constitutes an interesting exploration path.

Other technologies, such as selective laser melting or electron beam melting, may enable the manufacture of similar constructs, with detail in the same order of magnitude, using alternative adequate basis materials for in vitro and even in vivo trials, including Ti powder and other powders from different alloys, ceramics, and even polymers, depending on the final application.

An additional degree of precision, even down to details of hundreds of nanometers, can be obtained by using two-photon lithography, but currently only for parts in the μm^3^ range. The promotion of the final part size is challenging but necessary for applications interacting not just at the single cellular level, but with larger portions of tissues and even whole organs. Companies such as Stratasys, Realizer GmbH, SLM Solutions GmbH, and Arcam provide some of the most interesting machines for metallic additive manufacturing based on different approaches and energy sources.

Regarding two-photon polymerization, NanoScribe GmbH commercializes the most precise direct laser writing systems (the Photonic Professional series) currently available, although its medical applications linked to large implantable implants and tissular constructs would require larger manufacturing workspaces.

Concerning the manufacture of design-driven knowledge-based tissue engineering scaffolds, lattice structures, and functionally graded porous structures, lithography-based ceramic manufacture, commercialized by Lithoz GmbH, provides the highest available degree of precision for the manufacture of highly complex geometries using a wide set of (bio-)ceramic materials.

### 3.2. Validation ofa Three-Dimensional Design ofa Niche for Skeletal Scaffolds

We assessed whether the functionalized niche of carbon fibrils/fibers and 3D-printed polyamide could be adequate to promote the adhesion and colonization of hMSCs within 3D composite structures or niches. To this end, hMSCs were incubated, as indicated in the Materials and Methods Section, during 48 h using DMEM-LG–10% FBS medium, and the carbon fibrils and 3D-printed support frameworks were then individually placed in multi-well plates and tubes, respectively. Cells were fixed and carbon samples were used to analyze α-tubulin and nuclei, while the 3D-printed surfaces were observed using crystal violet dye.

**Figure 4:**
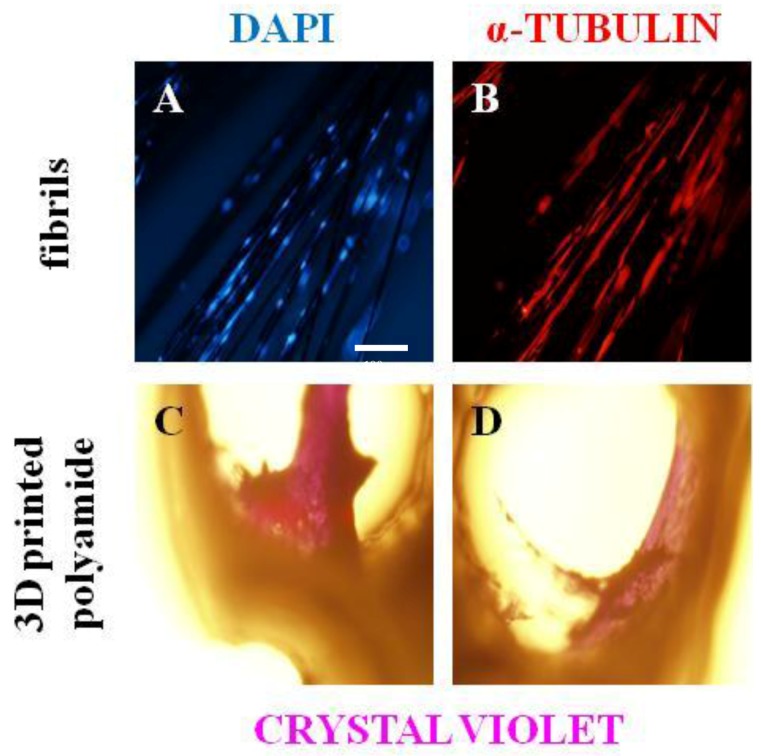
hMSC adhesion on 3D fabric polyamide–carbon fibril composite. (**A**) Fluorescence detection of hMSCs’ nuclei with DAPI. (**B**) Immunofluorescence determination with α-tubulin. Scale bar represents 100 µm. (**C**,**D**) hMSCs micro-masses dyed with crystal violet on 3D-printed polyamide.

An example of the results is shown in Figure 4. In Figure 4A,B, nuclei and α-tubulin, respectively, from hMSCs adhered on carbon fibrils. Interestingly, hMSCs recognize differences in the 3D polyamide surface with respect to the carbon fibrils and form micro-masses inside of the helicoidal 3D-printed structure (Figure 4C,D). In addition, the functionalized structure minimizes the floatability and movement of carbon fibers in aqueous media and shows its excellent property for cell adhesion. Furthermore, it can be suggested, by the degree of polymerization of tubulin, that hMSCs can migrate along the fibers. The 3D-printed structure gives permeable and biocompatible support to the carbon fibril niche that can be adapted and extended to specific mechanic properties and geometries needed to tailor and to personalize skeletal tissue scaffolds.

It is also well recognized that cells respond to surface roughness by modification in cell adhesion, growth, or differentiation. Thus, we decided to investigate whether the 3D-printed composite, which was functionalized by hMSC-CM as earlier described, could alter the chondrogenic process of hMSCs. The former process is a wide distributed process involved in cartilage tissue and in the formation, remodeling, and repair of long bones of the axial skeleton and vertebrae. Consequently, we compared the chondrogenic differentiation process of 2 × 10^4^ bone-marrow-derived hMSCs under three different conditions.

**Figure 5:**
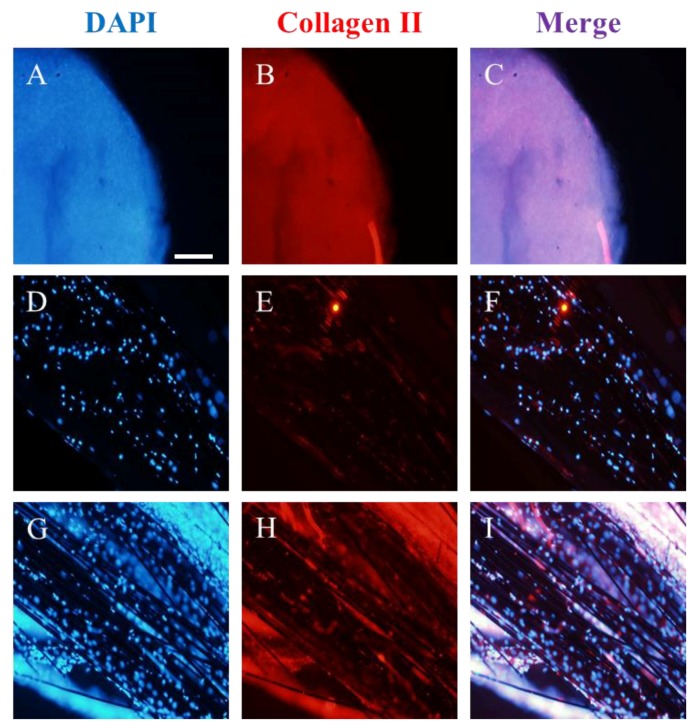
Induction of hMSC chondrogenesis on 3D-printed polyamide–carbon fibril composite by CM-hMSC functionalization. A quantity of 2 × 10^4^ hMSCs control micro-masses (**A**–**C**), or seeded on 3D-printed polyamide–carbon fibril composite (**D**–**F**), or seeded on 3D-printed polyamide–carbon fibril composite pre-treated with hMSC-CM (**G**–**I**). Blue represents hMSCs nuclei (DAPI) and red indicates immunodetection of collagen type II. Scale bar represents 50µm.

The chondrogenic process lasted a week and the results are summarized in Figure 5, showing the micro-masses used as control (Figure 5A–C); the 3D-printed composite niches (Figure 5D–F); and the 3D-printed composite niches functionalized with hMSC-CM (Figure 5G–I). Samples were studied in triplicate. As could be expected, control micro-masses became buried in collagen type II (Figure 5B), while carbon fibers had hMSCs adhered (Figure 5E,H). Clearly, the expression of collagen II, which helps to highlight the chondrogenic differentiation, was lower in the un-functionalized samples (E) than in the samples functionalized with hMSC-CM (H). In the functionalized samples, a collagen surface that resembles a glide-like knee cartilage can be seen [25].

Consequently, present works support that carbon fibrils and their functionalization using hMSC-CM provide a new tool for tissue engineering, in particular for specific scaffolds needed after skeletal tissue distraction. Although, additional bench work is needed to determine the exact mechanism involved in hMSC-CM, the elasticity and the porosity of specific pieces of skeletal tissues can be mimicked using the proposed approach.

**Figure 6:**
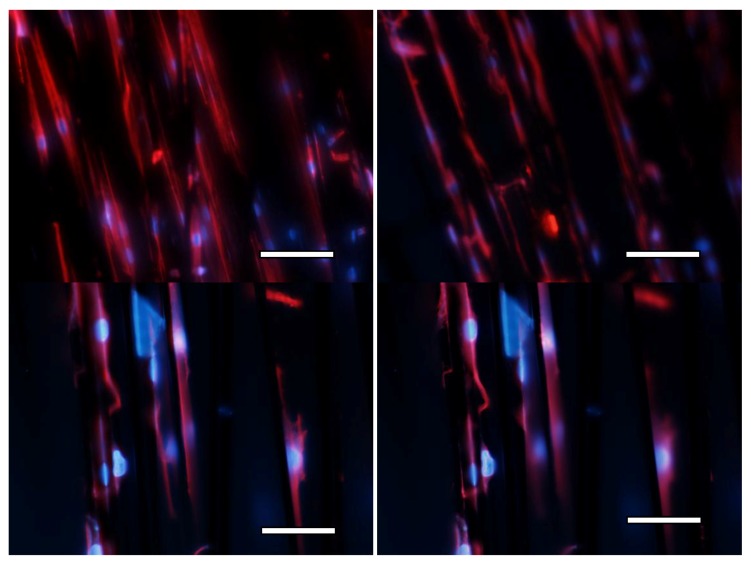
The cells attached and spreading upon the carbon fibrils functionalized with hMSC-CM show a good energetic behavior and adequate viability. Fluorescent dyes: α-tubulin (red for the cytoskeleton) and DAPI (blue for the nuclei). Upper images scale bar: 100 μm. Lower images scale bar: 200 μm.

Finally, in order to additionally assess cell viability and the energetic behavior of cells, a second set of trials was carried out, seeding again both phases of the scaffold, especially focusing on the interaction of cells with the carbon fibrils filling and labeling both the nuclei and the cytoskeletons, as previously detailed in the experimental section. Figure 6 helps to show the good energetic behavior of the cells. The images show several cells attached and spreading upon the fibrils and more than 30 expanded active cells can be seen per visual field in the low magnification images and more than 10 in the high magnification images. Additional studies, using improved confocal microscopy, may provide more details about the three-dimensional configuration of the cells within the whole construct, but we understand that the provided validation shows promising results.

## 4. Discussion

Our results show that the cells and the carbon-fiber-filled frameworks are excellent companions for potential tissue repair strategies. In case of tissue damage, the hMCS-seeded 3D-printed framework may constitute a personalized support, in order to allow the permeation of nutrients and of debris, to promote oxygenation, to enable adaptation, and to provide cellular communication systems capable of locally inhibiting the immune system and of activating tissue repair following the fluid dynamic; however, additional and more systematic assessments, both in vitro and in vivo, need to be performed before thinking about clinical application.

The performed experiments, even if limited by available materials and equipment, help to check the viability of using design-controlled 3D-printed frameworks for user-defined mechanical properties and potentially personalized three-dimensional features, as well as the adequate cell–framework interactions shown by the cell masses formed upon the laser-sintered scaffolds. The knitted fibers provide guidance for cell–cell unions and facilitate chondral differentiation, as shown by the liberation of synthesized collagen to the extracellular medium (Figure 5H).

The carbon fiber filler, especially when functionalized with hMSC-CM, may promote the scaffold’s colonization and final tissue viability, as has been also proven with other types of tissues (i.e., in solutions aimed at bone repair) [27]. Last but not least, the hMCS-seeded carbon-fiber-filled frameworks offer interesting possibilities to study cellular mechanisms present in different types of tissue, especially the interactions between different types of musculoskeletal tissue linked to articular repair. The cell–material interactions may be extended to a triad composed of (1) hMSCs, (2) carbon-fiber-filled frameworks, and (3) endoderm-/exoderm-derived cells for studies on complex tissues (i.e., for solutions linked to osteo-articular repair).

These results clearly differ from the cell morphologies and behavior shown in recent studies by our team, in which 2D and 2D½ approaches, relying in some cases on carbon materials, clearly affected cell morphology and, importantly, promoted mobility. In our case, three-dimensional colonization and chondral differentiation is seen [28,29].

Future studies will deal with more systematic in vitro and in vivo analyses for assessing the performance of the tubular scaffolds and of alternative constructs, including annular lattices, as well as biomimetic constructs with functional gradients of properties, such as those presented in the computer-aided design section; these biomimetic constructs may also provide interesting solutions in the field of tissue repair and regeneration, especially in the area of articular damage and reconstruction, perhaps as a complement for softer tissues to already available effective solutions for bone repair based on composites and nano-composites [30,31].

The computer-aided designs presented here may be improved, in terms of biomimicry, by using recently proposed strategies based on the use of multi-morphology transition hybridization CAD design of minimal surface porous structures [32]. The irregular features of living tissues can be also taken into account and modeled by means of multi-scale approaches [33] and by resorting to stochastic design procedures [34].

As a complement to our present study, additional combinations of fibers and nanofibers may also provide interesting results in terms of biomimicry [35] and their incorporation to 3D-printed constructs, possibly with a structure sintered from more adequate powders including nanoparticles or nanofibers and with the longer fibers knitted to the printed framework; such constructs may help to solve some current geometrical limitations of electrospinning, for instance. Potential synergies with recently developed processes linked to the three-dimensional printing of fiber-reinforced hydrogel composites may allow for the creation of more versatile tissue repair solutions covering a wider range of mechanical properties and helping with 3D colonization of the constructs [36].

Electrospun polymeric nanomats with nanocarbons [37], if applied upon 3D constructs, may also support cell guidance towards viable repair constructs. We believe that further research in these directions may lead to the promotion of micro-structural heterogeneity for directing micro-mechanics and mechanobiology [38], and that the presented combination is an interesting complement to already-discussed biomaterials and composite scaffolds for tissue repair [39,40], to 3D-knitted biomedical constructs [41,42], and to functionally graded and multi-material tissue repair solutions validated by means of MSCs [43,44].

## 5. Conclusions

Summarizing, in this study we have presented an innovative multi-material approach towards the straightforward, rapid, and low-cost development of biomimetic scaffolds for tissue engineering strategies, especially focusing on the potential repair of osteochondral tissues. The system is based on the additive manufacture (by selective laser sintering of polyamide) of three-dimensional computer-designed lattice structures or frameworks, into which carbon fibers are subsequently knitted or incorporated. In our work, the 3D-printed lattice structures have acted as supports for three-dimensional repair and cell growth and the knitted carbon fibers have performed as driving elements for helping with cell colonization of the repair constructs. A human mesenchymal stem cell (h-MSC) conditioned medium (CM) has been also used for improving the scaffold’s biological response and for promoting cell adhesion, proliferation, and viability, following trends in the biofunctionalization of tissue repair scaffolds. Cell culture results, during which scaffolds have become buried in collagen type II, have provided relevant information regarding the viability of the composite scaffolds used and the prospective applications of the proposed osteochondral repair strategy. In fact, the advanced composite scaffolds developed, together with the conditioned medium functionalization, constitute biomimetic stem cell niches with clear potential, not just for cartilage and endochondral bone formation and regeneration strategies, but also for tendon and ligament repair, as we expect to demonstrate in forthcoming in vivo studies in our progressive approach to medical practice.
